# The MEMIC is an *ex vivo* system to model the complexity of the tumor microenvironment

**DOI:** 10.1242/dmm.048942

**Published:** 2021-08-18

**Authors:** Libuše Janská, Libi Anandi, Nell C. Kirchberger, Zoran S. Marinkovic, Logan T. Schachtner, Gizem Guzelsoy, Carlos Carmona-Fontaine

**Affiliations:** Center for Genomics and Systems Biology, Department of Biology, New York University, New York, NY 10003, USA

**Keywords:** Tissue mimetics, Tumor metabolism, Tumor microenvironment

## Abstract

There is an urgent need for accurate, scalable and cost-efficient models of the tumor microenvironment. Here, we detail how to fabricate and use the metabolic microenvironment chamber (MEMIC) – a 3D-printed *ex vivo* model of intratumoral heterogeneity. A major driver of the cellular and molecular diversity in tumors is accessibility to the blood stream. Whereas perivascular tumor cells have direct access to oxygen and nutrients, cells further from the vasculature must survive under progressively more ischemic environments. The MEMIC simulates this differential access to nutrients, allow co-culturing any number of cell types, and it is optimized for live imaging and other microscopy-based analyses. Owing to a modular design and full experimental control, the MEMIC provides insights into the tumor microenvironment that would be difficult to obtain via other methods. As proof of principle, we show that cells sense gradual changes in metabolite concentration leading to predictable molecular and cellular spatial patterns. We propose the MEMIC as a complement to standard *in vitro* and *in vivo* experiments, diversifying the tools available to accurately model, perturb and monitor the tumor microenvironment.

## Abstract (precis)

**Editor's choice:** We present how to fabricate the MEMIC, an experimental model of the tumor microenvironment, describing proof-of-principle experiments and providing image analysis tools that are helpful when using this system.

## INTRODUCTION

**Fig. 1.**
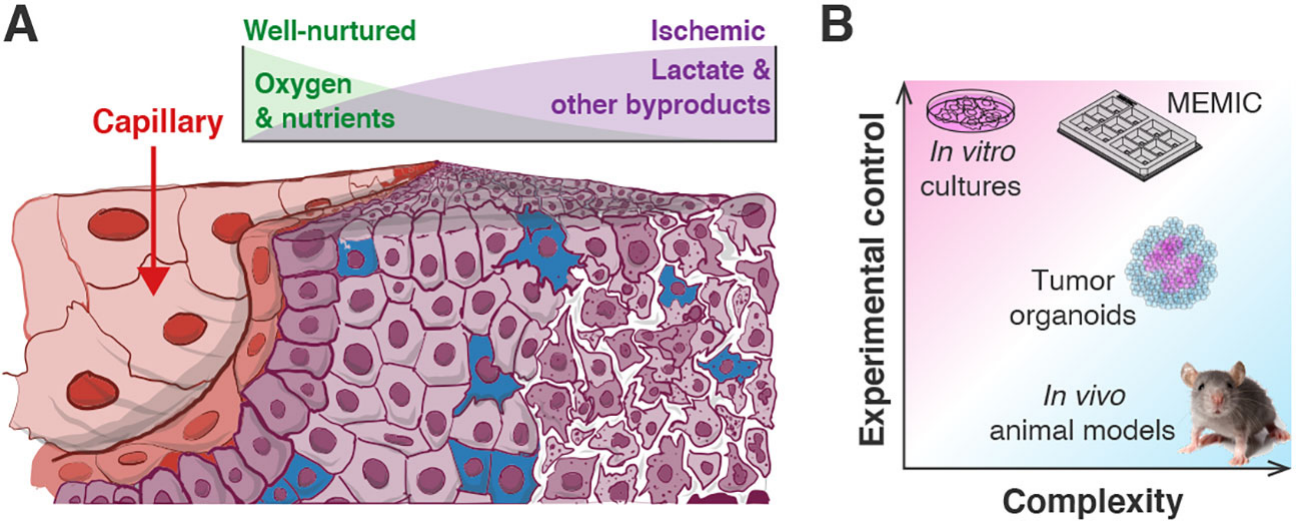
**The complexity of the tumor microenvironment and its experimental models.** (A) Owing to poor vascularization, tumor cells are exposed to different metabolic conditions according to their distance to blood vessels. Proximal cells readily obtain oxygen and nutrients, whereas distal cells endure a lack of resources and accumulation of metabolic byproducts. (B) Models of the tumor microenvironment. *In vitro* cultures provide a high level of experimental control, but they cannot capture key features of the tumor microenvironment. The complexity of *in vivo* models – and to some extent of 3D organoid cultures – comes at the cost of experimental control. The MEMIC allows for high complexity *in vitro* and *ex vivo* cultures while allowing for full experimental control.

The tumor microenvironment is a complex cellular ecosystem (Fig. 1A). This complexity includes a large diversity of malignant and non-malignant cells such as fibroblasts and immune cells (Junttila and de Sauvage, 2013; Merlo et al., 2006; Tabassum and Polyak, 2015). These cells secrete and consume molecular signals, growth factors, and metabolites, creating an intricate biochemical landscape in the tumor interstice that in turn affects tumor cell behaviors and cell–cell interactions (Bader et al., 2020; Buck et al., 2017; Mehta et al., 2017; Wiseman and Halliwell, 1996). Insufficient tumor vascularization can produce predictable spatial patterns of cell phenotypes. Cells located near functional blood vessels are constantly perfused with nutrients and oxygen. By contrast, cells distant from vasculature must survive in an environment that it is poorly nourished and profuse in potentially toxic metabolic waste products (Carmeliet and Jain, 2000; Gatenby and Gillies, 2004; Hobson-Gutierrez and Carmona-Fontaine, 2018; Thomlinson, 1977). The role that nutrients and other metabolites have in modulating the behavior and phenotypes of tumor – and especially immune cells – has been the subject of recent interest (Buck et al., 2017; Hobson-Gutierrez and Carmona-Fontaine, 2018; Olenchock et al., 2017; Pavlova et al., 2018). However, the lack of amenable models of the metabolic microenvironment of tumors hampers the ability to predict, control and treat the effects of environmental metabolites.

Animal models are a fundamental tool to study the complex and heterogeneous tumor microenvironment (Day et al., 2015; Gould et al., 2015). However, the complexity of animal physiology – although crucial in pre-clinical studies – can challenge the isolation of individual experimental variables, and their use for large experiments is severely limited by practical, economical and ethical concerns (Bert et al., 2017; Bressers et al., 2019). On the other side of the spectrum, conventional *in vitro* experiments offer much better experimental control and can be easily used in high-throughput approaches. However, these cultures do not model the metabolic heterogeneity and other essential features of the tumor microenvironment. The recent resurgence in the use of three-dimensional tumor organoids – or tumoroids *–* as a tool to model different aspects of tumor biology does offer some of these features (Clevers, 2016). Tumoroids can recapitulate key histopathological tumor characteristics, and they can be used to screen for patient-specific drug responses (Boj et al., 2015; Gao et al., 2014; van de Wetering et al., 2015). However, the organization of tumoroids emerges spontaneously, and thus the visualization, quantification and prediction of their organization remains challenging (Fig. 1B).

We previously developed a microphysiological system that mimics the complexity of the tumor microenvironment in a well-controlled and predictable manner. This metabolic microenvironment chamber (MEMIC) is suitable for high-resolution microscopy analyses and can be easily adapted to the complexity and throughput that different experimental scenarios may need (Carmona-Fontaine et al., 2017). Cells in the MEMIC are gradually limited in their access to fresh medium, generating gradients of extracellular metabolites and oxygen across the chamber in which they are cultured. This metabolic heterogeneity can be accompanied by the addition of other components of the tumor microenvironment, such as stromal cells, an extracellular matrix, and perturbations with carcinogens or drugs. Compared to the methods mentioned above, the spatiotemporal complexity that emerges in the MEMIC is predictable, reproducible and measurable.

Here, we expand on key features of the MEMIC and provide detailed guidelines on how to fabricate and use this system. We determined key parameters that shape metabolic gradients in the MEMIC, which we describe, alongside detailed information on how to assemble the platform, how to set up cultures of tumor cells – alone or in co-culture – and how to monitor these experiments using live imaging and fixed endpoint microscopy assays, such as immunofluorescence. We demonstrate that the MEMIC accurately captures the cellular response to nutrient and oxygen deprivation, and show that nutrient-deprived macrophages reduce epithelial features in neighboring tumor cells. Finally, we provide an image analysis pipeline designed to obtain information at the single-cell level from MEMIC images suitable for users without any coding experience.

## RESULTS

### MEMIC – an overview

A hallmark of the microenvironment of virtually all solid tumors is the presence of hypoxic and poorly nourished niches (Gatenby and Gillies, 2008; Hobson-Gutierrez and Carmona-Fontaine, 2018; Lyssiotis and Kimmelman, 2017; Thomlinson, 1977). These conditions are the result of the increased growth of tumor cells and insufficient blood perfusion (Baish and Jain, 2000; Carmeliet and Jain, 2000; Pavlova and Thompson, 2016). Because tumor growth and tumor vascularization are not uniform, they create a heterogeneous ‘metabolic microenvironment’ in which some cells experience near physiological conditions, whereas others endure severe ischemia, and potentially cell death, owing to lack of nutrients and accumulation of toxic waste (Carmona-Fontaine et al., 2013; Gatenby and Gillies, 2008; Thomlinson, 1977). The MEMIC is a 3D-printed microphysiological culture system specifically designed to model this spectrum of metabolic conditions (Movie 1). In addition, the MEMIC allows the co-culturing of any number of cell types to study how different cells interact and behave in different metabolic niches (Carmona-Fontaine et al., 2017).

**Fig. 2.**
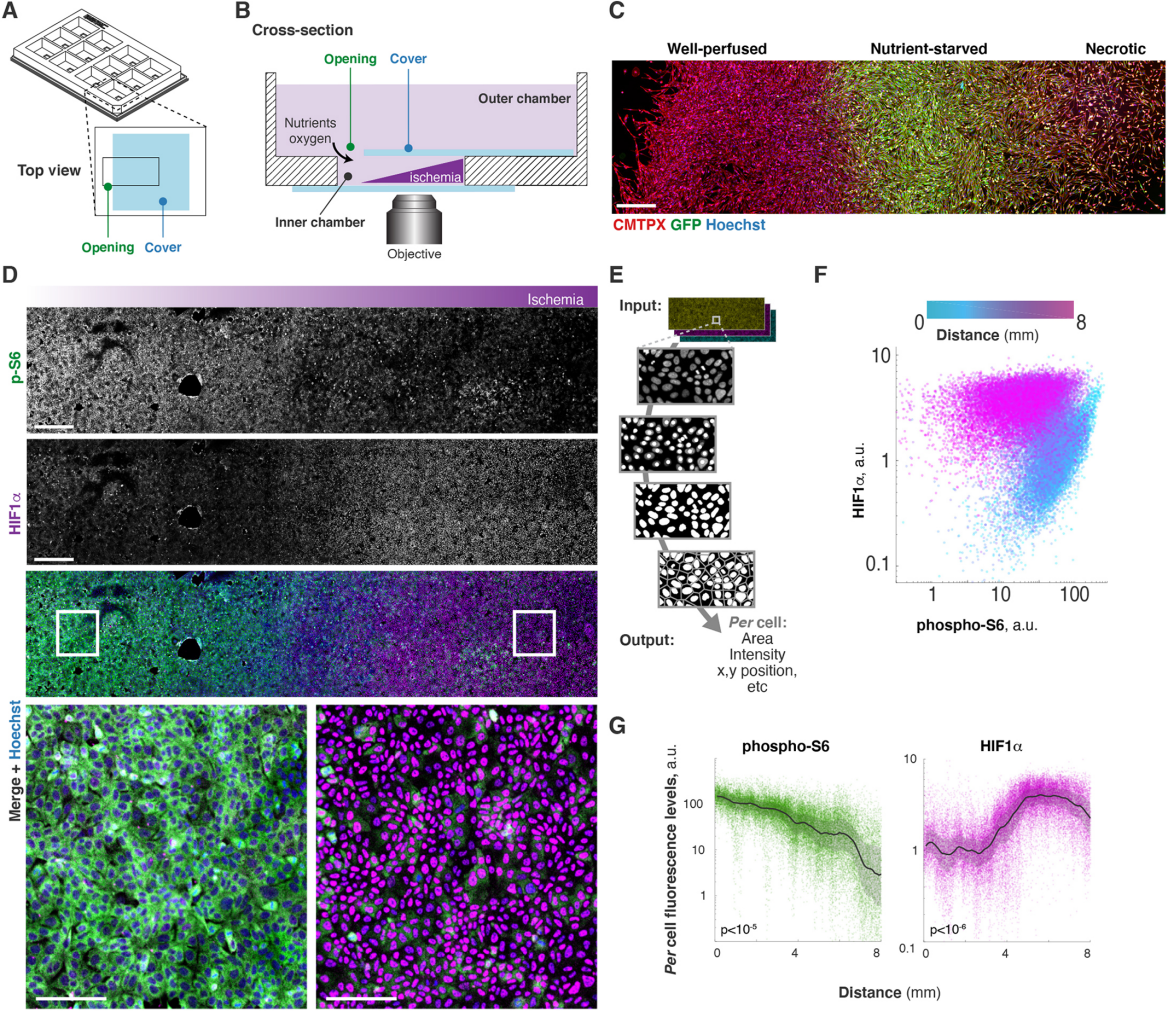
**Formation of metabolic gradients in the MEMIC.** (A) View of 3D-printed framework containing 12 independent MEMICs. Each chamber is located within a separate well. (B) Cross-section view of a MEMIC. Cells are cultured in the inner chamber, generating metabolic gradients. The chamber is enclosed by a top and bottom glass coverslip, except for a narrow opening to a media reservoir, allowing for unilateral diffusion of fresh medium into the chamber. (C) Cells expressing a GFP-based hypoxia reporter after 24 h culture in the MEMIC. Cells show distinct metabolic landscapes. Scale bar: 500 µm. (D) Metabolic conditions affect cell signaling and transcriptional programs. Immunofluorescent staining of DLD1 cells for phosphorylated ribosomal protein S6 (p-S6; a readout of active mTOR signaling, green) and for the transcription factor HIF1α (purple). Scale bars: 800 µm, 200 µm for insets. (E) A typical image cytometry pipeline. (F) Quantification of D, showing p-S6 and HIF1α levels per cell. Note the two distinct populations determined by the distance from the opening of the MEMIC. a.u., arbitrary units. (G) Quantification of D, showing opposite trends in intensity of p-S6 and HIF1α. Lines, moving median values; shaded regions, interquartile ranges. *P*-values were obtained by comparing data points from the first 10,000 points to the last 10,000 of the chamber using the rank-sum test.

To generate these gradients of metabolic conditions, cells in the MEMIC grow inside a small volume, or inner chamber, that connects from one end to a large reservoir of fresh medium (or outer chamber, Fig. 2A,B). The concentration gradients of extracellular metabolites in the MEMIC are established by a balance between their diffusion rate and their rates of consumption and secretion by cells in the inner chamber. Cells proximal to the opening, therefore, have a constant supply of nutrients and oxygen, and the metabolic byproducts they secrete are quickly cleared by fresh medium from the reservoir. By contrast, cells at the opposite end of the inner chamber endure a microenvironment in which oxygen and nutrients are scarce and metabolic byproducts accumulate (Fig. 2C).

The MEMIC framework described here is composed of 12 independent MEMIC chambers, facilitating multiplexing, technical replicates and no-gradient controls (Fig. 2A). The chamber in which cells grow is sealed by optical glass coverslips, and thus it is ideal for high-resolution microscopy analyses (Fig. 2B). The 12-MEMIC framework has the same footprint as a multi-titer plate, so it is compatible with conventional microscope stages and other equipment. The main requirement to fabricate a MEMIC is access to a 3D printer, which is inexpensive when compared to most laboratory equipment, and these are often available as a shared resource at universities and research institutions. Within the Supplementary Information linked to this article, we provide a detailed protocol (Fig. S1, Dataset 1) and a video (Movie 2) on how to 3D print and assemble this system.

### Cellular responses to nutrients and oxygen gradients

The mammalian target of rapamycin (mTOR) pathway is a major nutrient sensor and a regulator of anabolic metabolism (Saxton and Sabatini, 2017). We thus used readouts of this pathway to observe the cellular responses to nutrient gradients. We first cultured human colorectal adenocarcinoma cells (DLD1) in the MEMIC for 24 h and then used immunofluorescence to detect phosphorylated S6 (p-S6), a major target of the TORC1 pathway. For a detailed description on how to seed cells in the MEMIC and to process them for immunofluorescence, please refer to Fig. S2. Cells in these cultures show high levels of S6 phosphorylation near the opening of the MEMIC, but these levels decrease dramatically under ischemic environments (Fig. 2D). Conversely, hypoxia-inducible factor 1-alpha (HIF1α) is a transcription factor that regulates the cellular response to hypoxia. In the absence of oxygen, HIF1α is stable and accumulates in the nucleus, otherwise it is targeted for proteasomal degradation (Gordan and Simon, 2007; Semenza, 2013). In our MEMIC cultures, HIF1α is undetectable in well-nurtured cells, but its levels steeply increase in ischemic regions (Fig. 2D). A similar decrease in activation of the mTOR pathway (Palm et al., 2015) and increase in HIF1α (Zhong et al., 1999) has been observed in solid tumors *in vivo*.

To acquire information at the single-cell level, we wrote software that rapidly detects cell nuclei, segments individual cells and obtains key parameters such as their position, area and fluorescence intensity (Fig. 2E). This approach is similar to flow cytometry in that it quantifies the fluorescence of individual cells, but, unlike flow cytometry, it retains critical spatial information. To help the wider research community use this image cytometry approach, we provide MATLAB scripts and a step-by-step tutorial fit for scientists without any coding experience (see Dataset 2).

We used this image cytometry approach to analyze the images shown in Fig. 2D. We first plotted HIF1α levels for every cell versus its p-S6 levels (Fig. 2F). To incorporate spatial information, we color coded each cell on the plot according to its distance from the MEMIC opening. To emphasize the role of positional information and the spatial patterns that emerge, we can plot the fluorescence levels of each cell versus its distance from the opening of the MEMIC, as shown in Fig. 2G. The quantification shows an exponential decrease in mTOR activation as cells are further away from the opening, consistent with a gradual decrease in nutrient availability. By contrast, we observed a sharp increase in nuclear HIF1α within ischemic areas of the MEMIC. Although oxygen levels are expected to form a smooth gradient, we observed a stepwise increase in HIF1α levels. This digital response likely emerges from the threshold of oxygen tension that determines whether HIF1α is degraded or not (Lee et al., 2015; Wong et al., 2011). Overall, these results illustrate the formation of metabolite concentration gradients in the MEMIC and show that these gradients modulate intracellular signaling pathways in a predictable manner.

### Shaping metabolic gradients in the MEMIC

Interstitial levels of extracellular metabolites *in vivo* are determined by a balance of metabolite diffusion and cellular activities such as consumption and secretion rates. As such, the slopes of concentration gradients of these metabolites vary according to tissue crowding, nutrient transporter levels, growth rate and other parameters that are difficult, or impossible, to untangle *in vivo*. Gradients in the MEMIC are formed by the same principles; however, in this system, we are able to tune parameters independently and shape these gradients according to specific experimental needs.

**Fig. 3.**
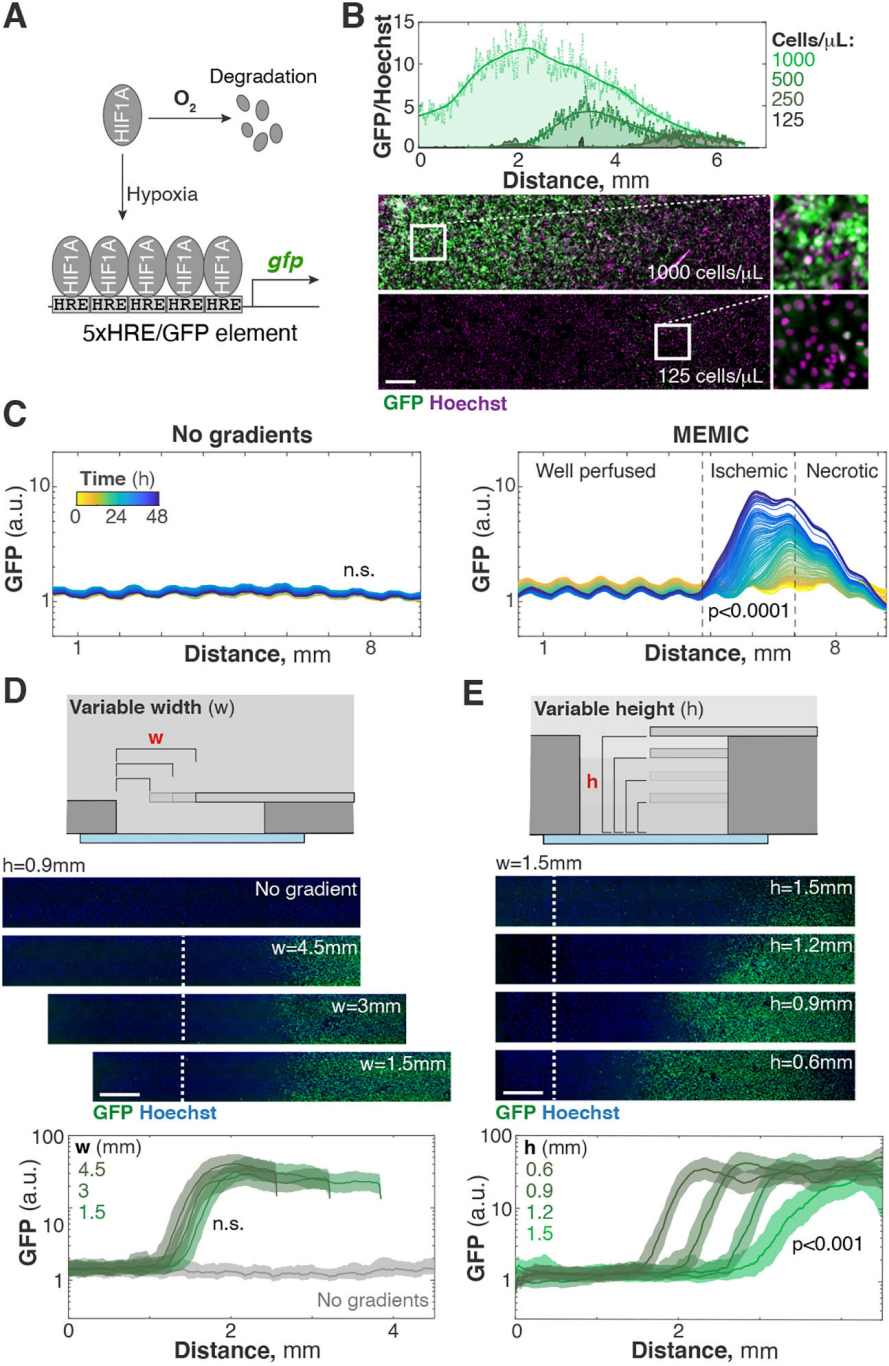
**Key parameters in shaping metabolic gradients in the MEMIC.** (A) We used a GFP-based hypoxia reporter (5xHRE-GFP) as a readout for resource limitation. (B) Cell density has a dramatic effect on the shape of metabolic gradients. Top: median GFP levels (normalized by Hoechst) for MDA-MB-231 cells expressing 5xHRE-GFP and cultured at different densities for 24 h in the MEMIC. Bottom: representative images of highest and lowest densities. Scale bar: 200 µm. (C) Monolayer of 5xHRE-GFP MDA-MB-231 cells shows that gradient formation in the MEMIC is dynamic and time dependent. Lines, moving median GFP intensity, color-coded by time. *P*-values were obtained by comparing population means from hours 1-10 to 39-48 using the rank-sum test. (D) 5xHRE-GFP MDA-MB-231 cells form gradients at the same distance from the opening and with the same shape when the width of the MEMIC opening is changed. Lines, moving median GFP intensity; shaded regions, interquartile range. *P*-values were obtained by comparing the locations in which populations achieved 50% of maximal GFP signal using a one-way ANOVA (no gradients condition was excluded from analysis). Each population estimate was obtained from ∼10^5^ cells, and three population replicates of each treatment were used in these comparisons. Lines, moving median GFP intensity; shaded regions, interquartile range. Scale bars for D and E: 500 µm. *P*-values were obtained by comparing the locations in which populations achieved 50% of maximal GFP signal using a one-way ANOVA. n.s., not significant. Each population estimate was obtained from ∼10^5^ cells, and three replicates were used to compare treatments.

To accurately measure and tune parameters in the MEMIC, we used a fluorescent hypoxia reporter as a readout for metabolic gradients (Vordermark et al., 2001). We engineered human breast tumor cells (MDA-MB-231) to express GFP under the control of five HIF1α-binding sites (Fig. 3A). As a fluorescence reference, we also expressed a membrane-bound form of mCherry in these cells (mCherry-CAAX).

Denser cultures should form steeper gradients as more cells consume nutrients and oxygen. To test the effect of cell density on these gradients, we seeded different cell numbers into the MEMIC and measured GFP levels after 24 h. With the lowest cell density (125 cells/µl or ∼12% confluency), GFP signal was not detectable, indicating that the collective oxygen consumption was not enough to activate a hypoxic response, even in the far end of the chamber (Fig. 3B). At higher cell densities, the GFP signal became clear, with steeper intensity gradients, larger amplitude and peaking closer to the opening. At the highest cell density (1000 cells/µl or >100% confluency), GFP levels peaked almost at the opening of the MEMIC (Fig. 3B). From there, GFP levels dropped as cells become anoxic and necrotic (Fig. 3B). Together, these results confirmed that denser cultures produce steeper metabolic gradients. In subsequent experiments, we used intermediate cell densities (250-500 cells/µl) that created strong gradients but that were shallow enough to clearly distinguish well-nurtured, nutrient-deprived and necrotic regions within the MEMIC (e.g. Fig. 2C).

The formation of the ischemic gradient is a dynamic process. To establish how these gradients develop over time, we seeded the same engineered MDA-MB-231 cells into the MEMIC and tracked their fluorescence levels using live microscopy and image analysis. Whereas constitutive membrane mCherry levels remained constant (Movie 3), GFP levels dramatically increased after ∼12 h in the distal regions of the MEMIC (Fig. 3C; Movie 3). Over time, the amplitude of the gradient increased and became steeper. In a control culture in which cells were well nurtured throughout the well, no hypoxia was detectable throughout the entire experiment (Fig. 3C).

We expected that the intensity and shape of metabolic gradients also depend on the geometry of the MEMIC chamber. Using mathematical modeling, we predicted that the height of the MEMIC is the critical geometric parameter in determining the position and slope of metabolic gradients (Carmona-Fontaine et al., 2013). This model also predicts that the width of the MEMIC's opening should not affect the shape of the gradient. To test these predictions, we seeded a constant number of MDA-MB-231 cells expressing the 5xHRE-GFP hypoxia reporter in MEMICs with different heights or with different opening widths and imaged them after 24 h (5×10^5^ cells, Fig. 3D,E). As predicted by our model, changing the width of the opening did not affect the shape of metabolite gradients. Cells seeded in MEMICs with different opening widths increased GFP expression at a constant distance from the opening. When we plotted the GFP levels versus distance to opening, the signals from all experiments collapsed into a single curve regardless of the opening width used (Fig. 3D). Although the width of the MEMIC opening has little physiological relevance, it has an important experimental implication: the exact position of the top coverslip does not matter as long as the edge of the glass is used as a reference point. This observation makes the fabrication of MEMICs easier and more reproducible.

As shown in Fig. 3E, the height of the MEMIC chamber is indeed key in shaping ischemic gradients. Cells cultured in thin MEMIC chambers (short height) have less medium, resulting in steeper gradients that peak close to the opening (Fig. 3E). In taller chambers, ischemic gradients are shallower and peak further inside the chamber. The length scales in the MEMIC are much larger than *in vivo*, where cells are tightly packed, and, thus, their equivalent of the MEMIC height parameter is very small. As a reference point, tumor cells are completely hypoxic at ∼150-200 µm from the closest blood vessel (Carmona-Fontaine et al., 2017; Hobson-Gutierrez and Carmona-Fontaine, 2018; Thomlinson, 1977). This distance is about an order of magnitude smaller than gradients in the MEMIC and spans only ∼10-20 cell diameters. The shallower gradients in the MEMIC stretch spatial patterns to a few hundred cell diameters, greatly increasing the spatial resolution of our experiments. Despite this being an advantage for our experiments, it is important to keep in mind these scale differences and adapt the MEMIC's geometry if needed.

### Effects of metabolic gradients on cell proliferation

**Fig. 4.**
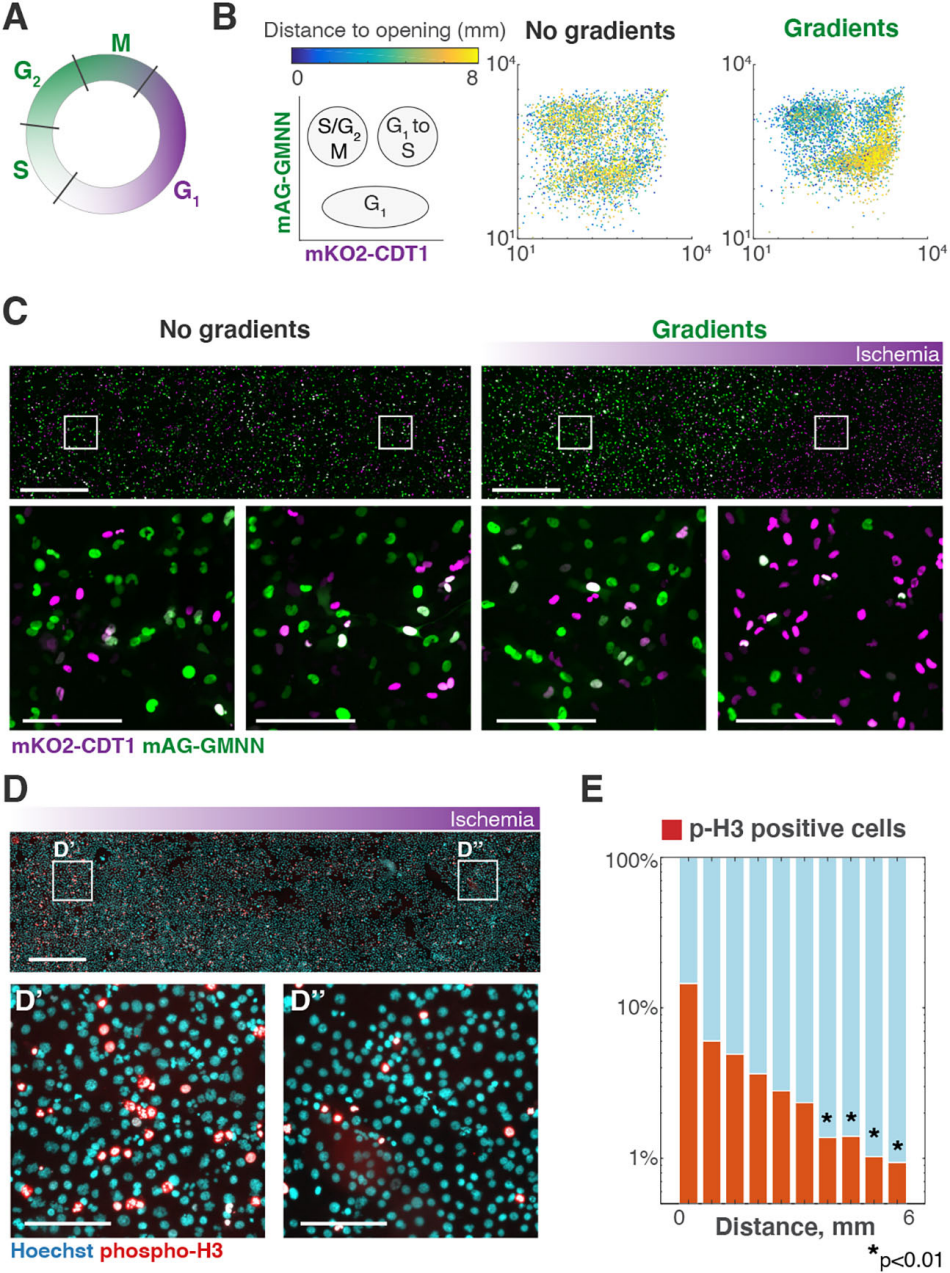
**Gradients of ischemia induce spatial patterns of cell proliferation.** (A) Relationship between mKO2-CDT1 (purple) and mAG-GMNN (green) fluorescent levels and cell cycle phases for cells expressing fluorescent ubiquitination-based cell cycle indicator (FUCCI). (B) Quantification of FUCCI fluorescence intensity in each RPE1 cell nucleus. Cells arrested in G1 are much more abundant in ischemic regions. (C) Representative images of RPE1 cells expressing FUCCI culture without gradients or in the MEMIC. Scale bars: 1000 µm, 200 µm for insets. (D) Immunofluorescent staining of phospho-H3 (red) in lung adenocarcinoma cells cultured in the MEMIC. Scale bars: 1000 µm, 100 µm for insets. (E) Quantification of D, showing the percentage of phospho-H3 (p-H3)-positive cells binned along the distance from the opening. The percentage of proliferating cells drops in ischemic regions. *P*-values were obtained by comparing the percentage of phospho-H3-positive cells in each bin versus the percentage of phospho-H3-positive cells in the first bin using unpaired Student's *t*-test. Data were obtained from 12 randomly acquired visual fields, each one containing at least 100 cells.

Nutrients provide the biomass and energy that all cells require to proliferate, and tumor cells are no exception. Cells in perivascular, and other well-perfused tumor regions, proliferate actively, whereas cells within nutrient-deprived tumor regions often show lower growth rates, senescence and can ultimately die (Carmona-Fontaine et al., 2013; Gatenby and Gillies, 2008; Thomlinson, 1977). To test whether we can reproduce these spatial patterns of cell proliferation in the MEMIC, we used cells expressing fluorescent ubiquitination-based cell cycle indicator (FUCCI) (Fig. 4A), a dual fluorescent reporter that enables the visualization of cell cycle progression (Sakaue-Sawano et al., 2008). Using our image cytometry approach (Fig. 2E), we quantified fluorescence levels per nuclei. To avoid confounding effects due to the aneuploidy and polyploidy that most tumor cells have, we used RPE1 cells – a nearly diploid immortalized epithelial human cell line with a relatively stable genome (Davoli and de Lange, 2011; Watkins et al., 2020). RPE1 cells proliferate rapidly, as shown by the many cells in M phase in the no-gradients control culture (Fig. 4B). The same was true in well-nurtured regions of the MEMIC. However, ischemic cells showed a stark arrest at G_1_, denoted by high mKO2-CDT1 and low mAG-GMNN levels (Fig. 4C). A similar arrest at G_1_ has been reported in tumors *in vivo* (Yano et al., 2014). *De novo* nucleotide synthesis is very sensitive to nutrient limitation (Ducker and Rabinowitz, 2017), suggesting that ischemic cells in the MEMIC – and in tumors – may arrest in G_1_, as they fail to synthesize enough nucleotides to duplicate their genomes. We detected a similar spatial pattern of cell proliferation in tumor cells using immunofluorescence. Lung adenocarcinoma cells proliferate rapidly, as evidenced by a high number of nuclei positive for phosphorylated histone H3 (p-H3). As shown in Fig. 4D, these numbers dramatically decline in ischemic regions of the MEMIC (Fig. 4D,E). Overall, these results show that metabolic gradients in the MEMIC induce changes in cell proliferation that are consistent with nutrient availability and *in vivo* observations.

### Untangling the effects of different metabolites in the MEMIC

The fast proliferation rate of tumor cells requires exogenous sources of multiple nutrients including glucose (Gatenby and Gillies, 2004; Vander-Heiden et al., 2009), aspartate (Birsoy et al., 2015; Sullivan et al., 2015), non-essential amino acids such as glutamine (Son et al., 2013), glycine (Jain et al., 2012), serine (Maddocks et al., 2013; Possemato et al., 2011) and asparagine (Gatenby and Gillies, 2004; Vander-Heiden et al., 2009), as well as diet-derived essential amino acids, vitamins and lipids (Bose et al., 2020). Ischemic tumor cells *in vivo* experience reduced levels of oxygen and all the aforementioned nutrients – as well as an accumulation of secreted metabolic byproducts such as lactate. Modeling this complex environment is difficult, and, thus, most cell metabolism studies focus on limiting a single substrate.

**Fig. 5.**
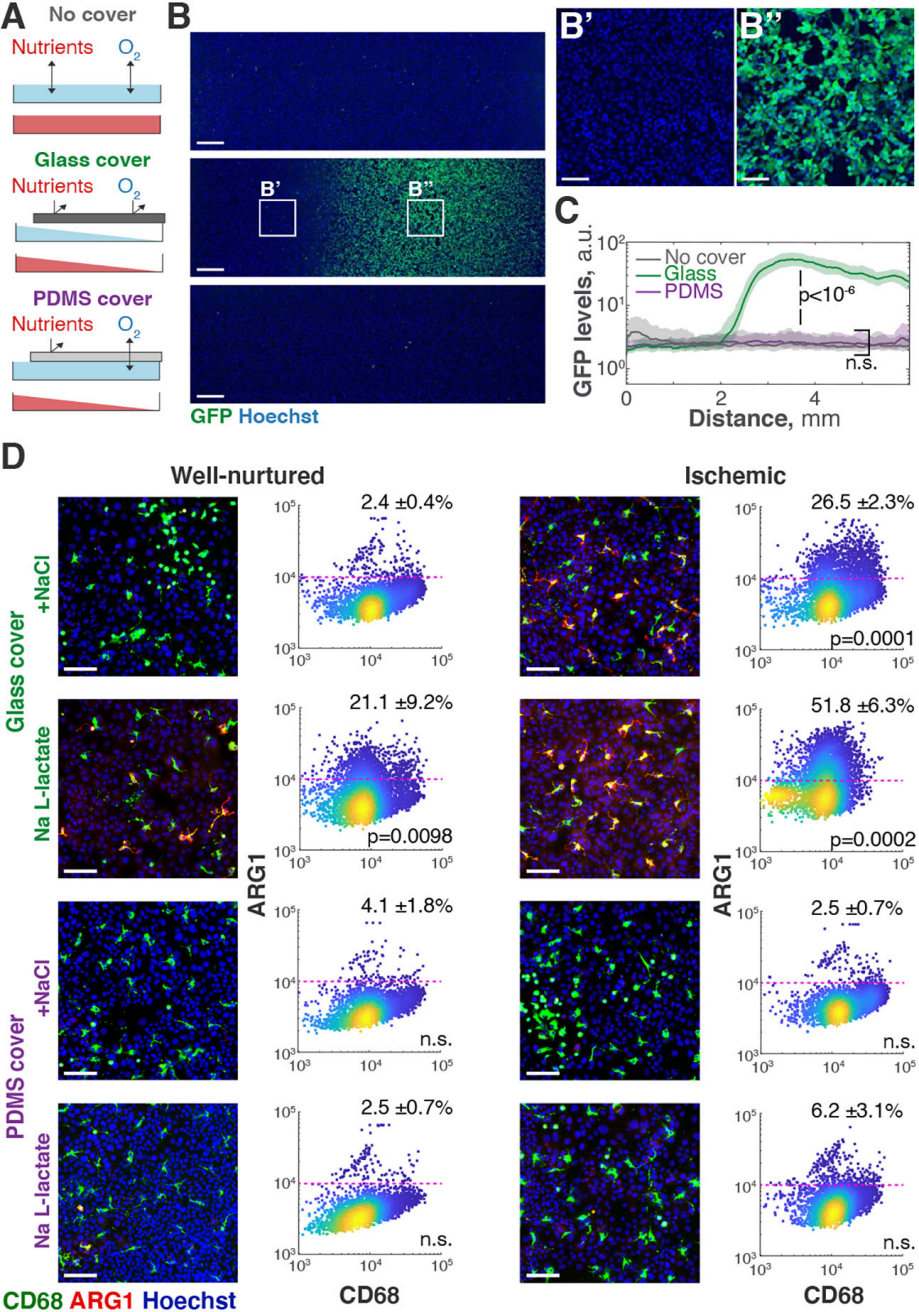
**Untangling gradients of hypoxia from other metabolites.** (A) Schematic showing the different experimental conditions depending on the use of glass or polydimethylsiloxane (PDMS) coverslips in the MEMIC. PDMS is permeable to gases, but not soluble metabolites, decoupling the nutrient gradients from hypoxia. (B) 5xHRE-GFP MDA-MB-231 cells grown in the MEMIC show hypoxia (GFP) in MEMIC with glass but not with PDMS coverslips (detail in B′ and B″). Scale bars: 500 µm for B, 100 µm for B′ and B″. (C) Quantification of GFP levels for all three conditions. Lines, moving medians; shaded regions, interquartile ranges. *P*-values were obtained by comparing the locations in which populations achieved 50% of maximal GFP signal using a rank-sum test. (D) CD68 versus ARG1 plots at the single-cell level and representative immunofluorescence images. Lung adenocarcinoma cells were co-cultured with BMDMs in MEMICs with glass or PDMS covers and treated with sodium lactate or NaCl for 24 h. We focused on well-nurtured and ischemic MEMIC regions. Percentages on the top correspond to the proportion of macrophages expressing high ARG1 levels (defined as twice the median levels in control macrophages). All *P*-values are in comparison with control condition (well nurtured, NaCl, in glass MEMIC) (unpaired Student's *t*-test). n.s., not significant. Data were obtained from nine randomly acquired visual fields, each one containing at least 100 cells.

In the MEMIC, cells spontaneously form complex metabolic gradients, better approximating *in vivo* conditions. Although these more physiological conditions can be advantageous, they can also be more difficult to separate the effects of individual metabolites and how they interact. In the MEMIC, however, we can easily distinguish effects that require hypoxia from those produced solely by nutrient scarcity. To decouple these factors, we take advantage of the gas permeability of polydimethylsiloxane (PDMS) (Cox and Dunn, 1986). PDMS is a biocompatible and transparent polymer that we can shape into membranes the size of the conventional glass coverslips that we use in the MEMIC (Fig. S1). Cells in MEMICs sealed with a PDMS membrane – instead of a glass coverslip – will experience gradients of nutrients, but no hypoxia, as oxygen is rapidly equilibrated across the PDMS membrane (Fig. 5A). To illustrate this approach, we cultured cells expressing the 5xHRE-GFP hypoxia reporter in MEMICs constructed with glass, PDMS or no coverslips (Fig. 5A). As shown in Fig. 5, only cells in a MEMIC sealed with glass showed GFP signal gradients (Fig. 5C). To test whether hypoxia is sufficient to produce a particular effect, these MEMIC experiments can be complemented with conventional *in vitro* cultures in hypoxic incubators in which oxygen is set at specific tensions. Similarly, to determine the effects of specific soluble nutrients, we can simply remove them from the media formulation, or we can add them at saturating concentrations.

These two approaches – using PDMS membranes to avoid hypoxia and changing media formulations – can be used together to untangle the effects of different metabolites. For example, we previously showed that a combination of hypoxia and high lactate is necessary and sufficient to increase ARG1 levels in macrophages (Carmona-Fontaine et al., 2017). We decided to use the MEMIC to reproduce this effect in a fully *ex vivo* setting. For macrophages, we used primary murine bone marrow-derived macrophages (BMDMs). To obtain freshly derived tumor cells, we injected mice with our 5xHRE-GFP/mCherry-CAAX lung adenocarcinoma cells. After 2 weeks, we removed the tumors and cultured the resulting clones. As shown in Fig. 5D, ischemic macrophages in the MEMIC dramatically increased ARG1 levels. The addition of lactate to the medium further increased ARG1 levels in ischemic macrophages and even in some mildly ischemic BMDMs. However, lactate had no significant effect on ARG1 levels when macrophages were cultured under normal oxygen levels, such as in MEMICs with PDMS membranes (Fig. 5D, bottom row).

### Ischemic macrophages decrease epithelial features in neighboring tumor cells

**Fig. 6.**
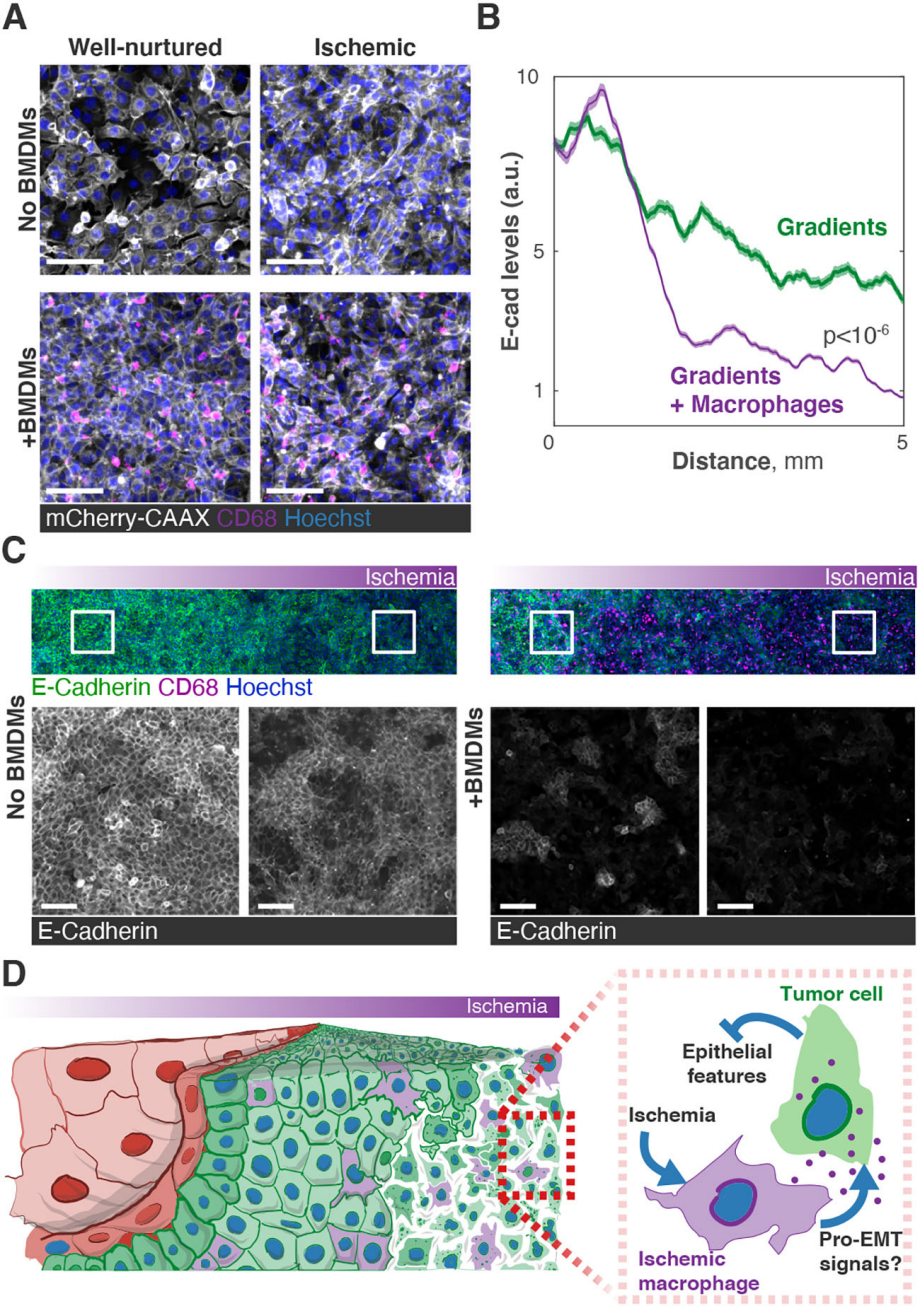
**Non-cell-autonomous effects of ischemia.** (A) Effects of ischemia on the morphology of tumor cells and these effects are accentuated in the presence of BMDMs. Scale bars: 50 µm. (B) Quantification of A, showing that the presence of macrophages synergizes with ischemic conditions to suppress E-cadherin expression. Lines, moving medians; shaded regions, interquartile ranges. *P*-values were obtained by comparing E-cadherin values at the last 1.5 mm of the MEMIC (using 4545 data points corresponding to ∼10,000 cells), and statistical significance was estimated using a rank-sum test. (C) Left: cells derived from MMTV-PyMT breast tumors exhibit decreased E-cadherin expression (green) in ischemic areas of the MEMIC. Scale bars: 100 µm. Right: cells derived from MMTV-PyMT breast tumors co-cultured with bone marrow-derived macrophages (BMDMs; CD68 immunofluorescence, magenta) show a stronger decrease in E-cadherin levels. Scale bars: 200 µm. (D) Schematic of a model in which ischemia and tumor-associated macrophages synergize to reduce epithelial features in tumor cells. EMT, epithelial-to-mesenchymal transition.

The complexity of the microenvironments within the MEMIC can be increased as needed. For instance, we can incorporate additional cell types to approximate the *in vivo* microenvironment and to study cell–cell interactions under different metabolic conditions. We observed that virtually every ischemic tumor cell cultured in the MEMIC became more dysplastic, lost their multicellular epithelial arrangements, and adopted a round or spiked morphology (Fig. 6A). These effects were exacerbated when tumor cells were co-cultured with BMDMs (Fig. 6A). We thus hypothesized that ischemia and the presence of macrophages synergize to alter the morphology of tumor cells.

To test this idea, we used clones derived from MMTV-PyMT breast tumors that normally present strong epithelial features, forming geometric epithelial structures, and display high levels of E-cadherin (Fig. 6B,C). Consistent with the known effects of hypoxia, we observed a significant decrease in E-cadherin (Fig. 6B) and a loss of epithelial organization (Fig. 6C) in ischemic cells. We compared these results with similar experiments in which tumor cells were co-cultured with BMDMs, ensuring that we maintained the same total cell number. In these co-cultures, ischemic tumor cells dramatically reduced their epithelial morphology (Fig. 6A) and the drop in E-cadherin was much steeper (Fig. 6B,C).

## DISCUSSION

*In vivo* animal tumor models are – and will continue to be – crucial in cancer research. However, they are expensive and difficult to scale up. Additionally, it is virtually impossible to accurately control and measure multiple experimental parameters in these experiments. At the other extreme of the experimental spectrum, conventional *in vitro* cell cultures allow the control of most experimental parameters, but this simplicity comes at the cost of losing the context of the intricacy and heterogeneity of tumors. The microphysiological culture system described here – named MEMIC – seeks to bridge these extremes by re-creating key aspects of the complexity of tumors under tunable and controllable conditions.

Cells in the MEMIC form concentration gradients of oxygen and nutrients equivalent to those formed in tumors *in vivo*. These different levels of nutrients induce changes in major signaling pathways that in turn change cell behaviors and phenotypes in predictable spatial patterns. As an example, we showed how concentration gradients of nutrients in the MEMIC lead to a heterogeneous microenvironment that ranges from well-nurtured conditions – in which cells rapidly proliferate – to increasingly more ischemic environments in which cells show cell cycle arrest. This heterogeneous distribution of resources and the resulting regions with high and low cell proliferation are well documented in the analysis of histological tumor samples from patients (Weinberg, 2013).

The simplicity and affordability of the MEMIC makes it compatible with mid- to high-throughput experiments and screens. Most chemotherapy drugs target rapidly proliferating cells, whereas senescent and non-proliferating tumor cells are spared, leading to lower tumor regression and relapse (Gordon and Nelson, 2012; Oshima et al., 2020). Thus, the MEMIC could be particularly useful for screening drugs that more efficiently target ischemic regions of tumors in which non-proliferating cells are abundant.

Because nutrient levels change gradually with distance from their source, cellular changes in the MEMIC follow predictable spatial patterns. We and others have previously shown that similar spatial patterns emerge *in vivo* following the distribution of the vascular network (Carmona-Fontaine et al., 2017; Gatenby and Gillies, 2008; Hobson-Gutierrez and Carmona-Fontaine, 2018; Thomlinson, 1977). The idea that nutrient and oxygen availability influence cell behavior and the tumor microenvironment is not new. However, this effect has been hard to model experimentally. With the combined power of *in vivo* observation and experiments in the MEMIC, we can argue that metabolite gradients do not only change cell phenotypes, but they organize and pattern the tumor microenvironment.

The spatial patterns that nutrient gradients impose in the tumor microenvironment also modulate cell–cell interactions. We showed here that ischemic macrophages reduce epithelial features in neighboring tumor cells. This inhibition of epithelial features is much stronger than the direct effects of nutrient deprivation alone. We thus propose that signals from ischemic macrophages synergize with the effects of hypoxia and nutrient deprivation (Fig. 6D). These morphological and molecular changes suggest that ischemic tumor cells may undergo epithelial-to-mesenchymal transition (EMT), a key step in the development of most carcinomas, which represent ∼80% of all solid tumors (Weinberg, 2013). Consistent with our observations, HIF1α directly promotes EMT (Dongre and Weinberg, 2019; Nieto, 2013) and tumor-associated macrophages can also drive EMT in tumors (Condeelis and Pollard, 2006; Linde et al., 2018). Although the molecular underpinnings of this synergy still need to be elucidated, these results illustrate how the MEMIC can be used to study the interplay between different cellular and molecular factors in the tumor microenvironment.

It is important to be aware of the limitations of the MEMIC. The system uses only a small number of cells (typically 10^4^-10^5^), which are insufficient for most biochemical approaches, such as genomics, transcriptomics and metabolomics. Furthermore, cells are ‘sandwiched’ between coverslips from which they are difficult to retrieve, especially without destroying the spatial structure of the system. These limitations are worth considering, especially because of the ever-growing interest in high-throughput quantification of nucleotide polymers. However, recent technological developments, such as imaging-based spatial transcriptomics methods like CODEX and STARmap (Goltsev et al., 2018; Wang et al., 2018), and spatially-resolved mass-spectrometry techniques (Dilillo et al., 2017; Passarelli et al., 2017), can offset some of the MEMIC's limitations.

Overall, the MEMIC provides a platform to model complex multicellular and heterogeneous *in vivo* conditions. In addition to having most of the advantages of conventional *in vitro* cultures, experiments in the MEMIC can be designed to incorporate high molecular and cellular complexity. This complexity can be increased gradually to carefully tease apart key factors shaping the tumor microenvironment. We believe that the process of deconstructing tumors into their basic units, and then carefully reassembling them in the MEMIC, will help us to better understand and control the tumor microenvironment.

## MATERIALS AND METHODS

### Cell culture

C6-HRE-GFP cells were a gift from Dr Inna Serganova (Memorial Sloan Kettering Cancer Center, New York, NY, USA). MMTV-PyMT cells were derived by Daniela Quail (McGill University, Montreal, QC, Canada). MDA-MB-231 (HTB-26), HEK293T, hTERT RPE-1 (CRL-4000) and DLD-1 (CCL-221) cells were purchased from American Type Culture Collection. All our cells are routinely tested for contamination and authenticated. All cells except RPE-1 were grown in high-glucose Dulbecco's modified Eagle medium (DMEM; Gibco, 11965-092) supplemented with 10% fetal bovine serum (FBS; Sigma-Aldrich, F0926). RPE-1 cells were grown in high-glucose DMEM-F12 medium (Gibco, 11039) containing 10% FBS. All cells were incubated at 5% CO_2_ and 37°C.

### Animal protocols and differentiation of BMDMs

We used male and female animals of 6-10 weeks of age. BMDMs were extracted from C57BL/6 mice using standard protocols (Gocheva et al., 2010). Following euthanasia, femurs and tibiae were harvested from both legs of the mice under sterile conditions. After careful cleaning of the tissue, the bone marrow was flushed using a 25-gauge needle and passed through a 40 μm strainer. The bone marrow flush was cultured in low-attachment culture dishes (VWR, 25384-342) in high-glucose DMEM supplemented with 10% FBS, 10 ng/ml Recombinant Mouse CSF-1 (R&D Systems, 416-ML), and 1× Antibiotic-Antimycotic (Gibco, 15240) for 7 days. Medium was replaced every other day to induce macrophage differentiation. All protocols involving animal work complied with relevant regulatory standards and were approved by New York University’s University Animal Welfare Committee (UAWC protocols 17-1496 and 19-1515).

### Construction of genetic fluorescent reporters

To create a hypoxia reporter for live imaging, an existing GFP-based HIF1ɑ reporter (Addgene, 46926; Vordermark et al., 2001) was subcloned into a lentiviral delivery plasmid using a Gibson assembly-based modular assembly platform (GMAP) (Akama-Garren et al., 2016). The 5xHRE-GFP region and a PGK-driven puromycin selection cassette from the pMSCV-Peredox-mCherry-NLS plasmid (Addgene, 32385) were amplified using primers containing overhangs with the homology sites for GMAP cloning and inserted into a lentiviral vector (LV 1-5; Addgene, 68411). This lentiviral backbone was a gift from Dr Tyler Jacks (Massachusetts Institute of Technology, Cambridge, MA, USA) and Dr Thales Papagiannakopoulos (New York University). The DNA construct for this new hypoxia reporter is available at Addgene.

To create a fluorescent membrane label, we used the CAAX sequence method to target a fluorescent protein to the plasma membrane (Hancock et al., 1989). mCherry was PCR amplified from pMSCV-Peredox-mCherry-NLS (Addgene, 32385) using primers containing overhangs with the homology sites for GMAP cloning. The reverse primer inserted the CAAX polybasic sequence. The CMV promoter and enhancer sequence to drive CAAX-mCherry expression was amplified from pLenti-CMV-Puro-DEST plasmid (Addgene, 17452). The two fragments were then assembled into one using overlapping PCR and inserted into LV 1-5 using the GMAP method. The DNA construct for this new fluorescent membrane reporter is available from Addgene as pLenti-mCherry-CAAX.

Both resulting constructs were delivered to destination cells through lentiviral transduction. Briefly, lentiviruses were packaged in HEK293T cells through co-transfection with 2 μg VSV-G, 4 μg Delta8.9 viral vectors and 6 μg 5xHRE-GFP or mCherry-CAAX vector using Lipofectamine 3000 reagent (Thermo Fisher Scientific, L3000) in Opti-MEM (Gibco, 31985). The vectors mentioned above were kindly provided by Dr Nicholas P. Gauthier (Dana Farber Cancer Institute, Boston, MA, USA). On day 2, Opti-MEM was replaced with fresh DMEM supplemented with FBS. On days 3 and 5 after transfection, the virus-containing medium was collected, filtered through 0.45 μm filters and immediately stored at −80°C until infection. For lentiviral infection, 1 ml freshly thawed virus medium mixed with 1 ml culture medium along with 10 μg/ml polybrene was plated onto destination cells in a six-well plate at 40-60% confluency. For the 5xHRE-GFP infection, infected cells were selected using 1 μg/ml puromycin for 4 days starting 48 h after transduction. For the mCherry-CAAX infection, infected cells were selected using fluorescence-activated cell sorting. To create the cell line with both mCherry-CAAX and 5xHRE-GFP, we used the lentiviral particles containing the mCherry-CAAX insert to infect a 5xHRE-GFP cell line and then selected for both using the above-mentioned selection methods. The same method was used to engineer cells to express the FUCCI sensor (Addgene, 86849).

### Printing and manufacturing the MEMIC

The main framework of the MEMIC is 3D printed using fused filament fabrication, which allows for on-demand supply as well as easy design modifications. For a detailed, step-by-step MEMIC fabrication protocol, please refer to Fig. S1 and Dataset 1, and to Movies 1 and 2. We designed the framework using computer-aided design software and printed it using Ultimaker 3D printers and black PLA filament (Ultimaker) – a biocompatible, non-autofluorescent and biodegradable material. Other printers and other materials may be used as well, depending on printer availability and experimental design. The printer settings, as well as the 3D design, that we use can be found in Dataset 1. We showcase a fused-filament-fabricated MEMIC version in this paper, as we believe that the combination of the widening availability of fused filament printers in research facilities and their precision would make this MEMIC the best option for most users. We also describe other options in Dataset 1.

Once a 3D-printed framework was retrieved from the printer, glass coverslips (No. 1, 18×18 mm; VWR) were glued to it using a UV-curable adhesive (NOA68, Norland Products) to create 12 individual chambers. We provide a detailed video on how to use the NOA68 and how to glue coverslips onto the MEMIC (Movie 2). To create the bottom of the chambers, the coverslips were glued so that one completely covers each opening. The glue must completely seal the glass surrounding the opening to prevent leakage from the bottom of the chamber but should not seep into the surface of the chamber in which cells will be seeded, as this limits space for cell adhesion. Once the adhesive was carefully applied, it was cured for 15 min under a long-wave UV lamp (DR-5401, MelodySusie). Uncured NOA68 is toxic for cells so UV exposure time can be extended if necessary. To complete construction of the inner chamber, glass coverslips were glued to the inside of each well with one edge against the inner ridge. This created the top layer of the gradient chamber and an opening to the media reservoir. Some chambers were left open without a top glass layer so that no gradients form, which are used as controls. The top layer adhesive was cured for 15 min under a long-wave UV lamp. Additional information and a detailed protocol on manufacturing the MEMIC can be found within Movie 2 and Dataset 1.

To decouple the nutrient and hypoxia components of the ischemic gradient in the MEMIC, in some experiments, the glass coverslip was replaced with PDMS coverslips. The PDMS coverslips were prepared using a Dow Sylgard 184 Silicone Encapsulant Kit. To prepare PDMS coverslips, one part of the curing agent was thoroughly mixed with ten parts of the silicone elastomer base by weight and centrifuged at 300 ***g*** for 2-5 min to remove any air bubbles. A Petri dish was then coated with ∼6 ml of the mixture by gently swirling it. To remove trapped air bubbles, the dish was placed into a vacuum desiccator for ∼5 min. Curing was done by placing the PDMS-coated dish into an oven at 75°C for at least 2 h. The layer of PDMS was then cut into 18×18 mm squares, matching the dimensions of the glass coverslip. Because PDMS is hydrophobic and may pose problems while gluing to the MEMIC framework, the PDMS coverslips were treated with a plasma gun. Then, the PDMS coverslips were glued with NOA68 optical adhesive as described for the top glass coverslips above.

### Alternative methods for MEMIC fabrication

The MEMIC framework can also be fabricated using stereolithography. This option provides similar benefits to fused filament fabrication, such as easy design modifications and on-demand manufacturing. In addition, fused filament fabrication of solid surfaces requires high printing precision as small errors can cause leaks through the framework, whereas stereolithography is more robust in building such surfaces. Stereolithography (SLA) printers are, however, typically less available as shared resources to most researchers. We use Form 3 (Formlabs) to print the same framework backbone as mentioned above using Formlabs's Dental SG resin. When using this approach, special care must be taken in thoroughly removing uncured resin, which is toxic to cells. To this end, we wash the prints in isopropanol for 1 h and we post-cure it with UV irradiation heated at 60°C. Following printing, washing and cleaning off the support structure, we glue the top and bottom coverslips as mentioned above. In Dataset 1, we also provide SLA-optimized MEMIC design.

For laboratories that cannot access 3D printers, we provide an option of MEMIC fabrication that does not require 3D printing and is therefore not flexible in design or as precise as the methods above. This procedure requires standard cell culture in six-well plates and a drill press. The drill press is used to drill a single 10 mm hole through each well of the six-well plate. This must be done carefully to avoid creating cracks or burn in the plastic. The new edges are then deburred. The bottom coverslips are glued first so that they completely cover the holes, similarly to the method described in the detailed protocol above. After curing the adhesive, the top coverslip is glued so that an opening is left from the created chamber to the remainder of the well. Opening size should be consistent between chambers. After curing the glue, this MEMIC framework can be used in experiments similarly to any other framework.

### Seeding cells into the MEMIC

To prepare the MEMIC for cell culture, the framework was first sterilized in a short-wave UV lamp (CM-2009, Meishida). Then, the inner chambers and wells of the MEMIC were washed twice with sterile Dulbecco's phosphate-buffered saline (DPBS; Gibco, 14040117). To improve cell attachment of some cell lines, the chambers were filled with a 50 μg/ml solution of poly-D-lysine (Sigma-Aldrich, P6407) in DPBS and incubated at 37°C overnight, then washed twice with DPBS. Afterwards, the chambers were washed with culture medium. Cells to be seeded were detached from their dish using 0.05% Trypsin-EDTA (Gibco, 25300-062). A cell suspension containing 10^6^ cells/ml was prepared, and the chambers were filled with 100 μl of the suspension. For co-cultures, we used the same total cell concentration and an initial cell ratio of 1:1. After 24 h, the number of tumor cells is higher as tumor cells proliferate faster than BMDMs. To open wells serving as controls, 500 μl of the cell suspension and 2.5 ml fresh medium was added. As the glass portion occupies about a fifth of the total surface of the well bottom, the resulting cell density on the glass portion should be similar to the closed wells. To allow cell attachment, the framework was placed into an incubator for ∼1 h. Once cells settled to the bottom of the chamber, the reservoir of the closed chambers was filled with 3 ml fresh medium. The framework was placed back into the incubator to start gradient formation.

### Immunofluorescence

After 24 h of gradient formation, the medium was aspirated from the wells and then carefully aspirated from the chambers using a pipette to prevent cell loss. The cells were fixed by filling the chambers with 2% (v/v) paraformaldehyde solution, obtained by diluting 4% paraformaldehyde (Affymetrix, 19943) 1:1 in PBS (Fisher Scientific, BP399) and incubating at room temperature for 5 min. The chambers were then gently washed twice with PBS. To permeabilize the cells for staining, the chambers were filled with a 0.1% (v/v) solution of Triton X-100 (Sigma-Aldrich, T8787) in PBS and incubated at room temperature for 5 min. The chambers were gently washed twice with PBS, filled with 100 μl blocking solution [2.5% (w/v) bovine serum albumin (Sigma-Aldrich, A9418) in PBS] and incubated for 1 h at room temperature. The chambers were gently washed twice with PBS. A primary antibody dilution was prepared in the blocking solution, with the antibodies diluted 1:200 for anti-HIF1α (BD Bioscience, 610958), 1:50 for anti-phospho-S6 (CST, 2211), 1:200 for anti-E-cadherin (BD Bioscience, 610182), 1:200 for anti-phospho-H3 (CST, 3377), 1:500 for anti-ARG1 (GeneTex, GTX109242) and 1:200 for anti-CD68 (Serotec, MCA1957), and added to the chambers. The framework was placed inside a humidified chamber at 4°C, and the primary antibody solution was removed. The chambers were washed three times with PBS and incubated at room temperature for 5 min. The chambers were then filled with a secondary antibody (1:500 dilution) and nuclear stain solution (Hoechst 33342, 1:2000) and incubated at room temperature for 1 h in the dark. The chambers were finally washed with PBS three times. To preserve cells until imaging, we filled the chambers with a 0.02% (w/v) solution of sodium azide (Sigma-Aldrich, S2002) in PBS.

### Microscopy

We imaged MEMICs using an inverted fluorescent microscope (Eclipse Ti2-E, Nikon) equipped with a spinning disk confocal unit (X-Light V2, CrestOptics). For live imaging, we used a stage top incubator (Tokai Hit, INUCG2-KRi) fed with a mixed gas source (5% CO_2_ in compressed air, Airgas, X02AI95C2000117). Entire chambers were imaged in different fluorescent channels using tiling microscopy and then exported as stitched 16-bit TIFF files.

### Image cytometry analysis

Microscopy images were processed using our MATLAB-based scripts as previously described (Carmona-Fontaine et al., 2017; Morris et al., 2019). A full tutorial on how to use our image cytometry code for users without image analysis experience is available with this paper (see Dataset 2 for further details). This analytic approach can be used with MEMIC images, and also with any other fluorescent images of cells or cell clusters for which fluorescence intensity and/or spatial information are of interest. Briefly, the software intakes images of multiple replicates or conditions, with a single TIFF image per channel per chamber, and outputs data on per cell fluorescence intensity, cell area on the image and cell position on the image. It also produces plots of fluorescence intensity versus distance for each channel. The image analysis process requires that at least one channel displays a uniform stain across, such as a nuclear stain for a cell monolayer.

### Methodology and statistics

In order to ensure that our sample size was allowed adequate statistical power, we conducted least three biological replicates for all experiments. We did not have a pre-established criterium to exclude samples from analyses. No particular method of randomization was used to determine how samples or animals were allocated to experimental groups, and investigators were not blinded to the group allocation. All statistical tests were two-tailed and non-parametric tests were used when data were not uniformly distributed.

## Supplementary Material

Supplementary information
