# Supplementary material for: The MEMIC is an ex vivo system to model the complexity of the tumor microenvironment
Source: Dis Model Mech. 2021 Aug 18;14(8):dmm048942. doi: 10.1242/dmm.048942 (PMC8382743; doi:10.1242/dmm.048942)
Supplement: Supplementary information [file dmm-14-048942-s1.pdf]

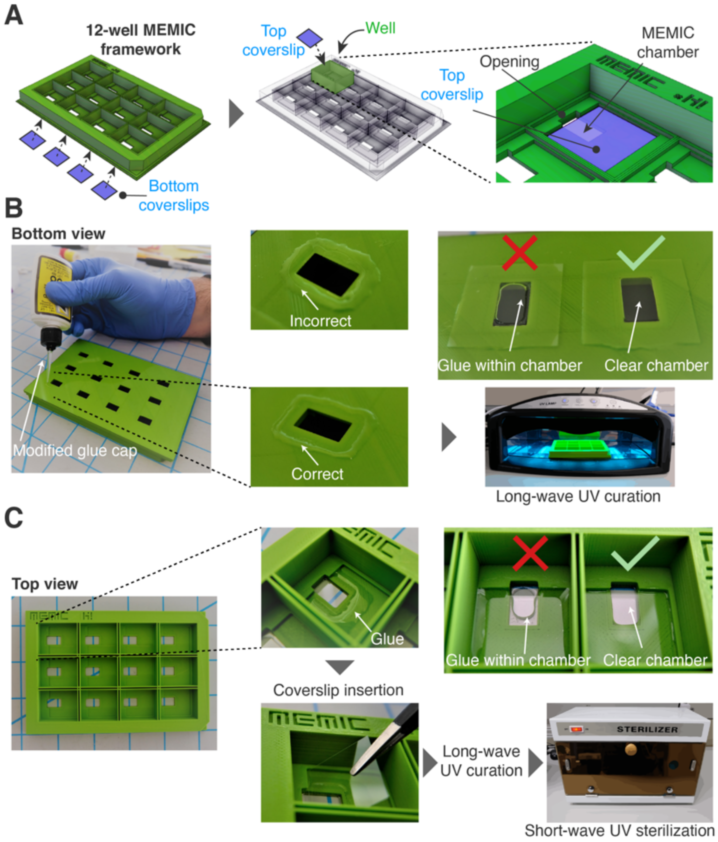

Fig. S1. Assembly of a 3D Printed MEMIC

A. The workflow of MEMIC assembly. First, coverslips are glued to the bottom of the 12-well MEMIC framework to create the surface on which cells grow (left illustration). In each well (see middle illustration), there is a small opening that becomes a MEMIC when a second coverslip is glued on top (right illustration). **B.** Bottom view of gluing the MEMIC. Apply a thin strip of glue about 3mm from the opening. Care should be taken not to apply too much glue or it will leak into the chamber impeding imaging and reducing the area for cell growth. The glue is cured by exposure to long-wave UV. **C.** Top view of gluing the MEMIC and coverslip insertion. Take care to not apply too much glue, to prevent leakage into the chamber. After curing with long-wave UV, the plates are sterilized using short-wave UV. Note that we normally use black PLA as a printing material. Green PLA is used in this figure for better image contrast.

Disease Models & Mechanisms • Supplementary information

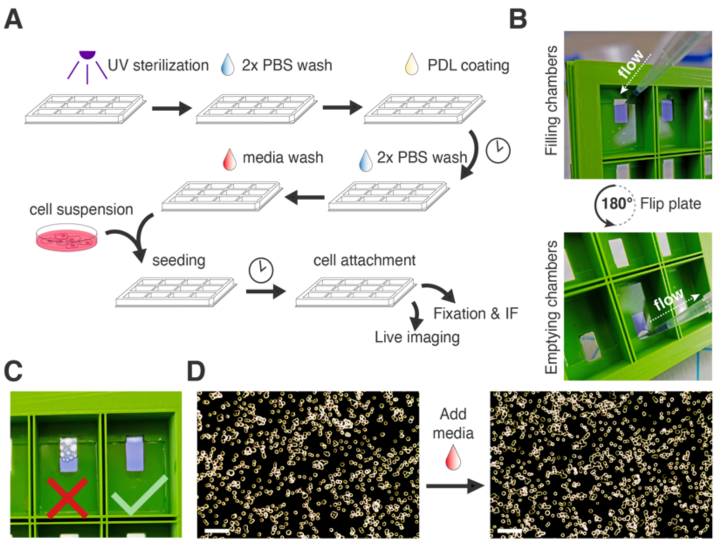

Fig. S2. Seeding and analyzing cells in the MEMIC

A. Typical workflow for a MEMIC experiment depicting the order of washing, coating and seeding steps. **B.** Process for filling and removing liquid from MEMIC chambers. When adding reagents, orient the MEMIC so that the slit is facing upward and pipette slowly from the corner under the coverslip. When withdrawing liquid, flip the MEMIC 180º so that the slit is facing downward and gravity aids in the removal process. **C.** Depiction of an incorrectly filled chamber and a correctly filled chamber. The incorrectly filled chamber contains several bubbles under the coverslip. **D.** DLD1 cells imaged immediately after seeding (left) and the same field imaged after media addition one hour later (right). Ideally adding media should not disrupt adhering cells. Scale bars: 50μm. Note that the second addition of media to the wells at this timepoint does not affect the homogeneous distribution of cells.

Disease Models & Mechanisms • Supplementary information

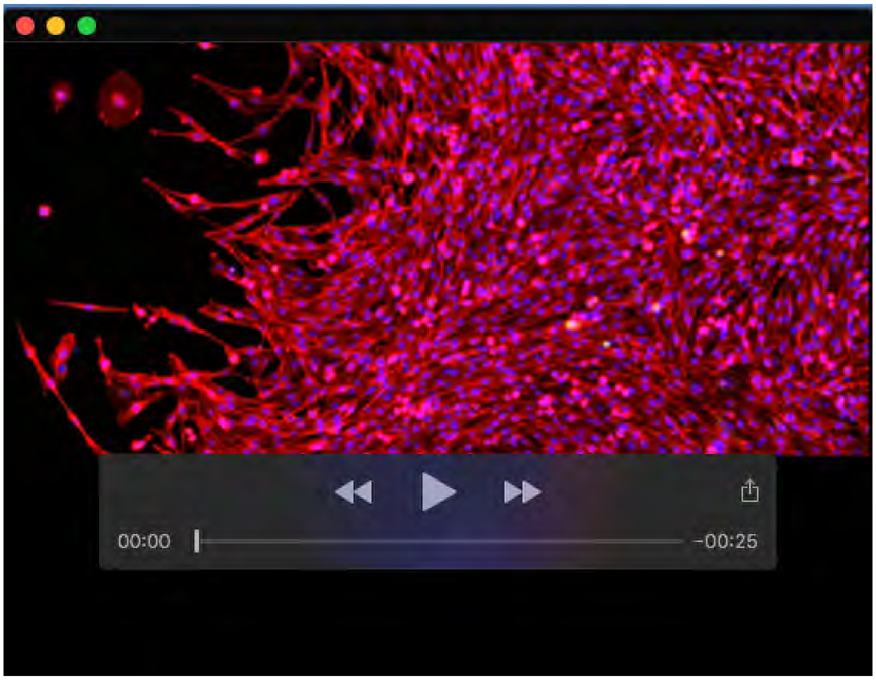

Movie 1. MEMIC design and printing procedure

Details of MEMIC design and its 3D printing process. The MEMIC contains 12 wells, each of them designed with an inner chamber (white opening) where cells are seeded under a coverslip (blue), which is connected through a small side opening to an outer chamber. For this video we used green PLA as the printing material for better contrast, but we normally use black PLA. The printing process takes 6-14 hours depending on 3D printer type and printing quality.

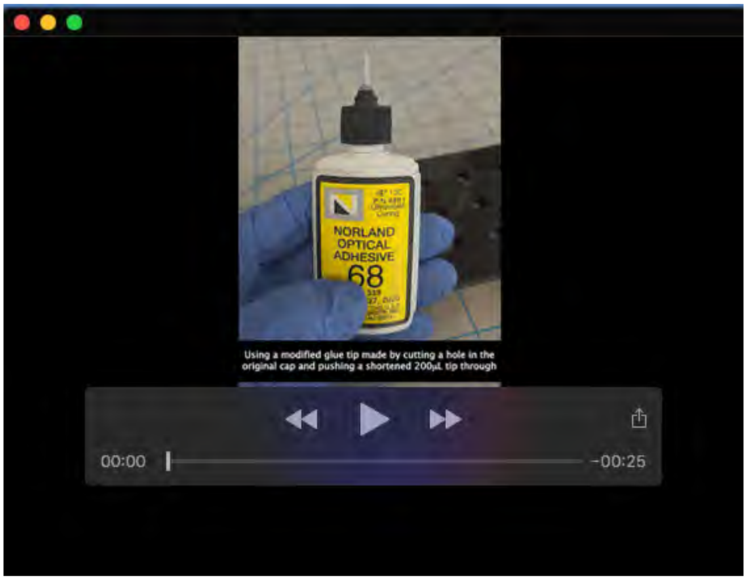

Movie 2. How to glue a MEMIC

A detailed video tutorial for gluing glass coverslips to the MEMIC framework.

Disease Models & Mechanisms • Supplementary information

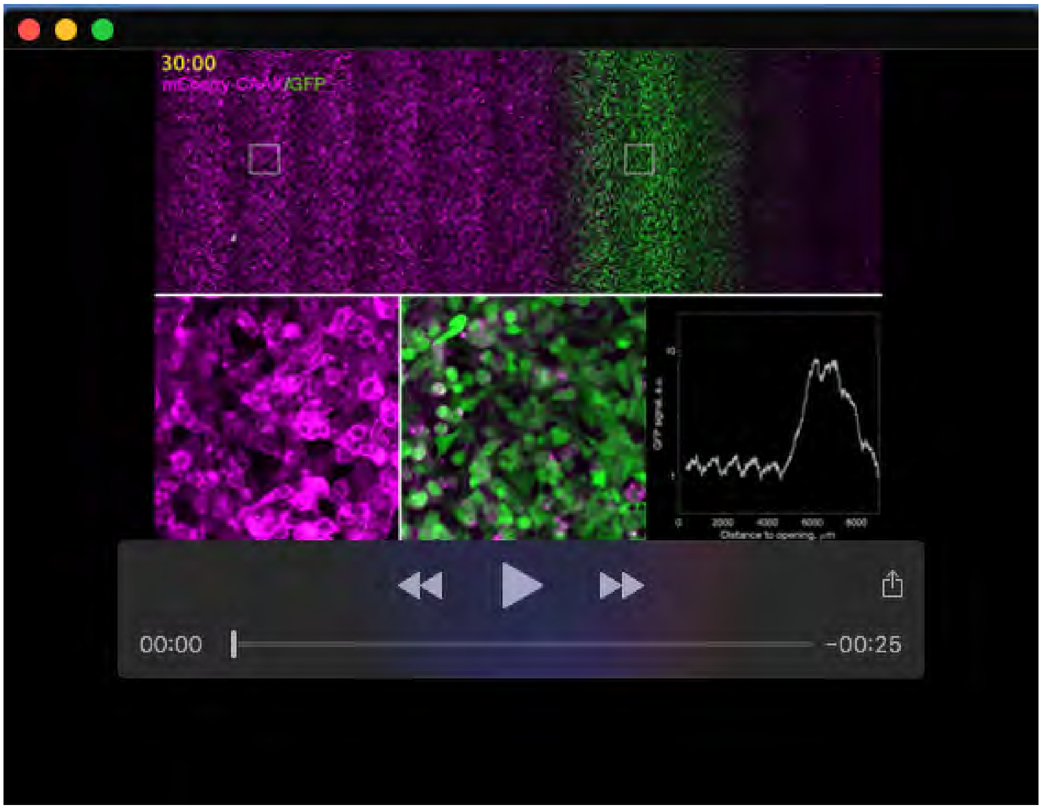

Movie 3. Emergence of oxygen gradients in MEMIC

Time-lapse of MDA-MB-231 tumor cells expressing membrane mCherry-CAAX and 5xHRE/GFP – a GFP-based hypoxia reporter. The MEMIC opening is on the left. The cells far from the opening demonstrate significantly higher levels of GFP than the ones near the opening at later time points. Quantification of GFP signal shows that oxygen gradients emerge within the MEMIC during cell incubation and increase over time. Total time: 32 h

Disease Models & Mechanisms • Supplementary information

Dataset 1. Compressed folder containing:

- 3D printing files:
  - MEMIC_2.0_FFP.stl
  - MEMIC_2.0_SLA.stl
- Detailed MEMIC protocols

Click here to download Dataset 1

Dataset 2. Compressed folder containing:

- MATLAB scripts:
  - Image_cytometer_test.m
  - Image_cytometer_quant.m
  - Image_cytometer_plot.m
  - otsu16bit.m
  - pkfnd.m
  - bpass.m
- Sample test and quantification images for the image cytometer scripts
- Excel sheet for variable input
- Image cytometry tutorial

Click here to download Dataset 2
